# Behind the Curtain: Prevalence of Symptoms of Depression, Generalised Anxiety and Eating Disorders in 147 Professional Dancers from Six Opera Houses or State Theatres

**DOI:** 10.1186/s40798-023-00638-9

**Published:** 2023-09-30

**Authors:** Astrid Junge, Anja Hauschild

**Affiliations:** 1 Center for Health in Performing Arts, Medical School Hamburg (MSH), Am Kaiserkai 1, 20457 Hamburg, Germany; 2 Center for Rehabilitation and Sports Medicine, BG Klinikum Hamburg, Hamburg, Germany

**Keywords:** Mental health, Risk factors, Sleep disturbance, Ballet, Performing artists

## Abstract

**Background:**

Mental health problems cover a wide spectrum. Depression and anxiety disorders are the most frequent mental health problem in the general population and in elite athletes. The aim of the present study was to assess the prevalence of symptoms of depression, generalised anxiety disorder and eating disorders in professional dancers, to compare the results between genders and to the general population and to analyse potential determinants.

**Methods:**

During a pre-season health screening, professional dancers of six German companies answered a comprehensive questionnaire on personal and dancer-specific characteristics, medical history and the Patient Health Questionnaire (PHQ-9), Generalised Anxiety Disorder Questionnaire (GAD-7) and Eating-Disorder-Examination-Questionnaire (EDE-QS).

**Results:**

A total of 82 (55.8%) female and 65 (44.2%) male dancers answered the questionnaire. One in five (20.8%) dancers had at least moderate symptoms of either depression, generalised anxiety disorder or eating disorders. The prevalence of at least moderate symptoms of depression was 11.1% in female and 6.4% in male dancers, of generalised anxiety disorder 16.0% in female and 6.4% in male dancers and of eating disorders 14.8% in female and 6.1% in male dancers. Compared to the general population of the same gender and similar age, the prevalence of at least moderate symptoms of generalised anxiety disorder was significantly higher in both genders. About one in four dancers (26.9%) reported a subjective need for support from a psychotherapist at the time of the screening. The PHQ-9, GAD-7 and EDE-QS sum scores were not related to the size or type of the companies, the age or rank of the dancers, but the PHQ-9 and GAD-7 sum score were significantly related to severity of musculoskeletal pain in the past seven days and to chronic or recurrent symptoms of low mood and generalised anxiety.

**Conclusion:**

The prevalence of symptoms of depression, generalised anxiety and eating disorders was high in professional dancers. Routine screening and low-threshold interventions to improve mental health of professional dancers are recommended.

**Supplementary Information:**

The online version contains supplementary material available at 10.1186/s40798-023-00638-9.

## Key points

- One in five (20.8%) dancers had at least moderate symptoms of either depression, generalised anxiety disorder or eating disorders, and more than a quarter of the dancers (26.9%) wanted or needed support from a psychotherapist/psychologist for personal or mental health problems at the time of the screening.
- The prevalence of at least moderate symptoms of depression was 11.1% in female and 6.4% in male dancers, of generalised anxiety disorder 16.0% in female and 6.4% in male dancers and of eating disorders 14.8% in female and 6.1% in male dancers.
- Mental health problems should be a part of routine health screenings of professional dancers, and low-threshold access to adequate treatment should be provided to dancers identified in screenings.

**Supplementary Information:**

The online version contains supplementary material available at 10.1186/s40798-023-00638-9.

## Background

Mental health of elite athletes has gained increasing attention in recent years [1, 2]. The International Olympic Committee (IOC) published a consensus statement on mental health in elite athletes to advance a more standardised, evidence-based approach to mental health symptoms and disorders in elite athletes [1] and recommended related assessment tools [3, 4]. While most reviews concluded that the prevalence of mental health problems of elite athletes is similar to non-athletes or the general population [1, 5–9], studies have also shown that the prevalence of depression, anxiety disorders and eating disorders varies substantially between different types of sport. [9–12]

Professional dancers are artists but also elite athletes considering the physical and mental demands of their training, rehearsals and performances [13, 14]. Similar to aesthetic sports, dancers must fulfil a specific body image and are often judged not only by their performance but also by their physical appearance. Further, dancers are exposed to various stressors that may have a negative impact on their mental health [15, 16]. These stressors have been categorised as dance cultural (e.g. physical ideals, normalisation of injury) [15, 16], intrapersonal factors (e.g. personality, health) [15], interpersonal factors (e.g. body image pressure, organisational relationships) [15, 16] and situational factors (e.g. career uncertainty, training load) [15, 16]. It has also been reported that dancers were exposed to more childhood trauma than athletes [17] and that dancers had a significantly higher distribution of post-traumatic stress disorders (20.2%) compared to the normal population [18]. This might explain why Thomson and Jaque [17] found that dancers experienced more anxiety and emotional difficulties than athletes.

Depression and anxiety disorders are the most frequent mental health problems in the general population [19] and in elite athletes [1, 2], but their prevalence has rarely been studied in professional dancers, whereas some information is available on dance students. Air [20] investigated the frequency of clinically significant psychological symptoms in 152 injured (professional, amateur and student) dancers visiting an orthopaedic clinic for musculoskeletal issues at their first or last visit. Sixty per cent of the dancers met the requirements for clinical referral to a psychologist or psychiatrist. Fostervold Mathisen et al. [21] found in their study on 124 dance students that 50% could identify personal mental health problems, 54% had depression and/or anxiety symptoms, and 12% had had an eating disorder diagnosed by a health professional. Van Winden et al. [22] monitored health problems in 130 dance students monthly during an academic year. Almost half (45%) reported a mental health problem, and 29% reported it as their most severe health problem. The most frequently reported mental health issues were depression, constant tiredness and stress due to external factors. Michaels et al. [23] reported that symptoms of depression and anxiety were common (35.4%) in a group of 198 collegiate dancers and that 37.4% of the dancers were interested in receiving mental health support. Liu et al. [24] observed a high prevalence of any co-occurring mood (47.1%) and anxiety disorders (30.9%) in dancer students with an eating disorder.

Several studies and a few reviews [25, 26] on eating disorders in dancers have been published. Arcelus et al. [26] concluded that the overall prevalence of eating disorders was 12.0% in dancers and 16.4% in ballet dancers. Keay et al. [27] found characteristics consistent with low energy availability (LEA) in 57% of 225 female dancers and 29% of 22 male dancers, indicating a risk of relative energy deficiency in sport (RED-S). A similar percentage of LEA in female dance students was reported by Fostervold Mathisen et al. [21].

In summary, it seems that mental health problems and especially eating disorders are frequent in (pre-)professional dancers. Therefore, the primary aims of the present study were (a) to assess prevalence of symptoms of depression, generalised anxiety and eating disorders in professional dancers, (b) to compare the prevalences in female and male dancers and (c) to compare the prevalences in dancers to the general population. The secondary aim was to analyse potential determinants of symptoms of depression, general anxiety and eating disorders.

## Methods

All dancers of six German opera houses or state theatres (*n* = 279) were asked to participate in a pre-season health screening prior the season 2022/2023. Two companies had less than twenty dancers, one company between twenty and fifty and three more than fifty dancers. Four companies danced primarily ballet, one company contemporary and one revue. The screening consisted of a medical examination, physical performance tests and a comprehensive questionnaire. Prior to the screening all dancers were informed about the content and aims of the study, and those participating gave their informed consent. The study has ethic approval (MSH 2021/137) of the Medical School Hamburg, Germany.

The questionnaire included questions on the socio-demographic and dance-specific characteristics, nutrition habits, subjective need and current attempt to lose or gain weight, medical history, current complaints, previous or current need of psychotherapeutic support and well-established questionnaires on depression, general anxiety and eating disorders (Additional file 1). The online questionnaire was pseudonymous (i.e. dancers used a personal code) to provide feedback on their individual health screening results to the dancers. Only the individual dancer and the last author of the study knew the match of code and name and kept this information strictly confidential and in accordance with the German data protection laws. No information about any individual physical or mental health screening result was given to anybody, except the dancers.

The depression module of the Patient Health Questionnaire (PHQ-9) [28] consists of nine symptoms whose frequency during the last two weeks should be rated on a four-point Likert scale (0–3). The sum score is used to categorise the severity as none or minimal (0–4), mild (5–9), moderate (10–14), moderately severe (15–19) or severe (> 19). PHQ-9 scores of 10 or more had a sensitivity of 88% and a specificity of 88% for major depression [28]. Age- and gender-specific references values from a nationally representative survey in Germany [29] were used for comparison results. The internal consistency (Cronbach's alpha) of the original version was 0.89 and 0.86 in different populations [28] and of the German version 0.87 [29].

The Generalised Anxiety Disorder-7 (GAD-7) [30] consists of seven symptoms whose frequency during the last two weeks should be rated on a four-point Likert scale (0–3). A sum score is calculated, and cut-off scores for mild, moderate and severe anxiety are 5, 10 and 15, respectively [30]. The authors reported a sensitivity of 89% and a specificity of 82% using a cut-off of 10 [30]. For comparison with a general population, age- and gender matched data from a representative German national survey (*n* = 5030) were used [31]. The internal consistency (Cronbach’s alpha) was 0.92 for the original [30] and 0.89 for the German version [31].

The Eating-Disorder-Examination-Questionnaire (EDE-QS [32]) consists of 12 statements whose frequency/intensity in the past seven days should be rated on a four-point Likert scale (0–3). A sum score is calculated with higher values indicating greater severity, and a cut-off of 15 was shown result in 83% sensitivity and 85% specificity [33]. The internal consistency (Cronbach's alpha) of the EDE-QS was 0.91 [32]. No representative survey using the EDE-QS in the general population was found in the literature for comparison.

All data were processed using Excel (version 16.74) and SPSS (version 27). Statistical methods applied were frequencies, descriptives, Cronbach's alpha, ANOVA, multivariate linear regression analysis, Chi^2^ and Phi test. Based on an expected prevalence of depression (female: 5.3%, male: 4.5%) [29], generalised anxiety (female: 7.4%, male: 3.2%) [31] and eating disorders (12.0%) [26], a precision of 0.05 and a population of 1300 dancers [34] (estimated 50% female) a minimum of 70 female and 45 male participants for depression, 91 female and 61 male participants for anxiety disorders and 145 participants for eating disorders should be included in the analysis [35]. According to Green [36] 120 subjects are needed for the multivariate linear regression analysis with 16 independent variables and a statistical power of 0.8 (beta = 0.2, alpha = 0.05, *R*^2^ = 0.07). Significance was accepted at *p* < 0.05. The level of significance for the analysis of potential determinants (Table 3) was *p* < 0.003 and for the multivariate linear regression analysis (Table 4) was *p* < 0.017 according to Bonferroni correction for multiple testing.

## Results

In total 147 dancers from four ballet (*n* = 107), one contemporary (*n* = 13) and one revue (*n* = 27) company answered the questionnaires during the screening prior the 2022/2023 season. Twenty-eight (19.0%) dancers were employed in companies with less than twenty dancers, thirty (20.4%) in a medium size company and 89 (60.5%) in companies with more than fifty dancers. The participation rate was on average 52.7% but varied substantially between companies: 42.6% in large, 76.9% in medium and 91.3% in small companies.

The 82 (55.8%) female and 65 (44.2%) male dancers were on average 27.1 years old (sd = 5.3, median 26.0, range 18–42 years) without difference between genders. All dancers had a professional dance education, were full time employed and had worked on average 4.5 years (sd = 4.0, median 4.0) at the current company. Seven dancers (4.8%) classified themselves as principal dancer, 33 (22.8%) as soloist, 94 (64.8%) as Semi-Soloist/Coryphées/Corps de Ballet, eleven (7.6%) as Eleve, and two did not answer this question.

### Prevalence of symptoms of depression and generalised anxiety disorder

The PHQ-9 and the GAD-7 were answered by 144 dancers (response rate 98.0%). About three quarters of the dancers reported no or minimal symptoms of *depression*, 25 (17.4%) mild, ten (6.9%) moderate and three (2.1%) moderately severe symptoms. The prevalence of moderate to severe depression symptoms was 11.1% in female and 6.4% in male dancers (Table 1). Neither the prevalence nor the mean sum score of depression symptoms differed statistically between genders.

**Table 1:** Prevalence of symptoms of depression and generalised anxiety disorder in female and male dancers and a general population similar age (25–34 years) and same gender [29, 31]

Symptoms	Female dancers	Female general population	Male dancers	General male population
Depression	*n* = 81	*n* = 351 [29]	*n* = 63	*n* = 279 [29]
None or minimal	56 (69.1%)	77.5%	50 (79.4%)	84.4%
Mild	16 (19.8%)	17.2%	9 (14.3%)	11.1%
Moderate	7 (8.6%)	4.4%	3 (4.8%)	2.5%
Moderately severe	2 (2.5%)	0.8%	1 (1.6%)	1.9%
Severe	0	0.1%	0	0.1%
Mean sum score (sd)	3.7 (4.0)	2.51 (3.01)	2.7 (3.3)	2.01 (3.24)

Symptoms	Female dancers	Female general population	Male dancers	General male population
Generalised anxiety	*n*= 81	*n* = 387 [31]	*n* = 63	*n* = 297 [31]
None or minimal	49 (60.5%)	70.2%	49 (77.8%)	76.4%
Mild	19 (23.5%)	22.4%	10 (15.9%)	20.4%
Moderate	7 (8.6%)	4.9%	3 (4.8%)	2.9%
Severe	6 (7.4%)	2.5%	1 (1.6%)	0.3%
Mean sum score (sd)	4.8 (5.0)	Not reported	3.0 (3.8)	Not reported

The age- and gender-specific prevalence of at least moderate depression symptoms of male (6.4% vs 4.5%) and female dancer (11.1% vs 5.3%) was statistically similar to the general population [29] (Table 1). For female dancers, the difference was slightly below statistical significance (Chi^2^ = 3.5, *p* = 0.06) and the mean sum score of depression symptoms was statistically higher than in the general population (t = 2.5; *p* < 0.01).

About two-thirds of the dancers reported no or minimal symptoms of *generalised anxiety disorder* (68.1%), 29 (20.1%) mild, ten (6.9%) moderate and seven (4.9%) severe symptoms (Table 1). The prevalence of at least moderate symptoms was about 2.5 higher in female (16.0%) than male (6.3%) dancers. This difference was slightly below statistical significance (Chi^2^ = 3.2; *p* = 0.07; Phi = − 0.15), but the mean sum score of generalised anxiety symptoms differed significantly between genders (Table 1; *F* = 5.8, *p* = 0.017).

When age- and gender-specific prevalences were compared, both female (Chi^2^ = 6.0, *p* = 0.01) and male (Chi^2^ = 6.3, *p* = 0.01) dancers had a higher prevalence of at least moderate symptoms of generalised anxiety symptoms than the general population [31] (Table 1).

### History of depression and anxiety problems

Asked for chronic/recurrent complaints, 15 (10.2%) dancers reported low mood, 39 (26.5%) general anxiety and 14 (9.5%) performance anxiety/stage fright. The gender difference was not significant for low mood and performance anxiety/stage fright, but twice as many female (*n* = 28, 34.6%) than male (*n* = 11; 16.7%) dancers reported chronic anxiety problems (chi^2^ = 6.0; *p* = 0.014; Phi = − 0.20). A quarter of the dancers had been diagnosed with “depression, anxiety, burnout or similar” either previously (*n* = 23; 16.9%) or currently (*n* = 11; 8.1%) without significant gender difference.

### Eating disorders, BMI, attitudes and behaviour related to weight and nutrition habits

All dancers answered the eating disorders questionnaire, and 16 (10.9%) dancers reported symptoms consistent with an eating disorder. Twenty (15%) dancers had been diagnosed with an eating disorder previously (*n* = 16; 12.0%) or currently (*n* = 4; 3.0%). Three dancers (75%) with a current and four with a previous diagnosis of an eating disorder were screened positive with the EDE-QS.

The BMI indicated an underweight (BMI < 18.5 kg/m^2^) in no male but in 33 (42.9%) female dancers, including nine (11.7%) with severe underweight (BMI < 17.5 kg/m^2^). The mean BMI of female dancers was 18.9 (sd = 1.4) and differed significantly between companies (range 18.3 to 20.4 kg/m^2^; *F* = 3.9, *p* = 0.004). At the screening about a third (*n* = 50; 34.5%) of the dancers thought they “should lose weight”, and more than a quarter (*n* = 41; 28.1%) “tried to lose weight”. Eighteen (12.4%) dancers thought they should gain weight, and 14 (9.6%) were currently trying to do so. More than a third of the dancers (*n* = 52; 35.4%) stated to “eat a special diet or exclude any foods”, and more than three quarters (*n* = 112; 76.2%) took food supplements, mainly vitamins, minerals and/or proteins. The comparison of female and male dancers is presented in Table 2.

**Table 2:** Comparison of female and male dancers with regard to symptoms of eating disorders (EDE-QS) [32], body mass index (BMI), attitude and behaviour related to weight, and nutrition habits

	Female dancers	Male dancers	Gender difference
**Eating disorders** (EDE-QS)	*N* = 81	*N* = 66	Chi^2^ = 2.9; *p* = 0.09 Phi = − 0.14
No (< 15)	69 (85.2%)	62 (93.9%)	
Yes (> 14)	12 (14.8%)	4 (6.1%)	
**Body mass index** (kg/m^2^)	*N* = 77	*N* = 65	*F* = 251.0; *p* < 0.001
Mean (sd)	18.9 (1.4)	22.5 (1.4)	
Minimum – maximum	15.4–25.5	19.8–25.1	
< 18.5	33 (42.9%)	0	
< 17.5	9 (11.7%)	0	
**Feeling you should …**	*N* = 79	*N* = 66	Chi^2^ = 21.5; *p* < 0.001 Phi = 0.39
Lose weight	37 (46.8%)	13 (19.7%)	
Gain weight	2 (2.5%)	16 (24.2%)	
Neither	40 (50.6%)	37 (56.1%)	
**Currently trying to…**	*N* = 80	*N* = 66	Chi^2^ = 15.9; *p* < 0.001 Phi = 0.33
Lose weight	28 (35.0%)	13 (19.7%)	
Gain weight	1 (1.3%)	13 (19.7%)	
Neither	51 (63.7%)	40 (60.6%)	
**Special diet**	*N* = 81	*N* = 66	Chi^2^ = 10.5; *p* = 0.001 Phi = − 0.27
No	43 (53.1%)	52 (78.8%)	
Yes	38 (46.9%)	14 (21.2%)	
**Food supplements**	*N* = 81	*N* = 66	Chi^2^ = 0.45; *p* = 0.50 Phi = 0.06
No	21 (25.9%)	14 (21.2%)	
Yes	60 (74.1%)	52 (78.8%)	
**Diagnosis of eating disorder**	*N* = 78	*N* = 55	Chi^2^ = 12.9; *p* = 0.002 Phi = 0.31
No	59 (75.6%)	54 (98.2%)	
Previously	15 (19.2%)	1 (1.8%)	
Currently	4 (5.1%)	0	

### Prevalence of at least one mental health problem

Fourteen (17.3%) female and five (7.9%) male dancers had moderate or severe symptoms of either depression or generalised anxiety disorder, eight (9.9%) female and three (4.8%) male dancers of both. One of the 16 dancers with an eating disorder had also moderate or severe symptoms of depression, two of generalised anxiety disorder and two of both. In total 22 (27.2%) female and eight (12.7%) male dancers had moderate or severe symptoms of either depression, general anxiety or eating disorders at the time of the screening.

### Need for psychotherapeutic support

More than 60% of the dancers stated that they “wanted or needed support from a psychotherapist/psychologist for personal or mental health problems” previously (*n* = 51; 35.2%) or currently (*n* = 39; 26.9%) without significant difference between male and female dancers. The percentage of dancers who needed professional help for personal or mental health problems at the time of the screening varied between companies from 6.7% (*n* = 1) to 37.5% (*n* = 9).

Almost 70% of the dancers with at least moderate symptoms of depression and 58.8% with at least moderate symptoms of generalised anxiety disorder but less than half (43.8%) of the dancers with an eating disorder (based on EDE-QS) and a third (3 of 9) dancers with a severe underweight (BMI < 17.5) stated that they wanted or needed psychotherapeutic support at the time of the screening.

### Determinants of depression, generalised anxiety disorder and eating disorders

Comparison of the mean PHQ-9, GAD-7 and EDE-QS sum scores of male and female dancers with different characteristics is presented in Table 3. For both genders, the *PHQ-9 and GAD-7* sum scores were significantly related to severity of musculoskeletal pain in the past seven days, chronic or recurrent symptoms of low mood and generalised anxiety as well as to a previous or current diagnosis of “depression, anxiety, burnout or similar” (Table 3). Male and female dancers with sleep problems had higher depression scores, but only male dancers with sleep problems had higher generalised anxiety scores. The mean sum scores of the three mental health questionnaires differed significant between groups with no, previous or current need for psychotherapeutic support only in female but not in male dancers. In both genders, the mean *EDE-QS* sum score was significantly higher in dancers who thought they should lose weight or currently tried to lose weight (Table 3). In female dancers the mean sum score differed also between groups with no, a previous or current diagnosis of an eating disorder. The mean PHQ-9, GAD-7 and EDE-QS sum scores were *not related* to the size or type of the companies, nor to the age or rank of the dancers.

**Table 3:** Comparison of the depression (PHQ-9) [28], generalised anxiety disorder (GAD-7) [30] and eating disorder (EDE-QS) [32] scores of male and female dancers with different characteristics

Variable	Female dancers			Male dancers		
	PHQ-9	GAD-7	EDE-QS	PHQ-9	GAD-7	EDE-QS
Category (*n* of female /* n* of male dancers)	Comparison Mean (sd)	Comparison Mean (sd)	Comparison Mean (sd)	Comparison Mean (sd)	Comparison Mean (sd)	Comparison Mean (sd)
**Size of the company**	*p* ≥ 0.050	*p* ≥ 0.050	*p* ≥ 0.050	*p* ≥ 0.050	*p* ≥ 0.050	*p* ≥ 0.050
< 20 dancers (16/12)						
20–50 dancers (15/14)						
> 50 dancers (50/37)						
**Type of dance**	*p* ≥ 0.050	*p* ≥ 0.050	*p* ≥ 0.050	*p* ≥ 0.050	*p* ≥ 0.050	*p* ≥ 0.050
Ballet (60/47)						
Contemporary (8/5)						
Revue (13/14)						
**Age of dancer**	*p* ≥ 0.050	*F* = 3.6; *p* = 0.033	*p* ≥ 0.050	*p* ≥ 0.050	*p* ≥ 0.050	*p* ≥ 0.050
18–24 years (30/14)		6.6 (6.2)				
25–29 years (28/29)		4.0 (3.7)				
30–42 years (23/20)		3.4 (3.8)				
**Rank of dancer**	*p* ≥ 0.050	*p* ≥ 0.050	*p* ≥ 0.050	*p* ≥ 0.050	*p* ≥ 0.050	*p* ≥ 0.050
Principal dancer/Soloist (20/19)						
Others (59/44)						
**Body mass index** (kg/m^2^)	*F* = 4.2; *p* = 0.018	*F* = 3.4; *p* = 0.039	*p* ≥ 0.050	n/a	n/a	n/a
> 18.5 (44/63)	2.8 (3.0)	3.8 (3.4)				
17.5–18.5 (24/0)	5.4 (5.0)	6.7 (6.6)				
< 17.5 (9/0)	2.6 (2.4)	3.3 (3.4)				
**Severity of musculoskeletal pain**^#^	**F = 10.0; p < 0.001**	**F = 10.0; p < 0.001**	*p* ≥ 0.050	**F = 16.9; p < 0.001**	**F = 8.3; p ≤ 0.001**	*p* ≥ 0.050
No or mild (0–3 NRS) (45/40)	2.3 (3.2)	2.8 (3.4)		1.5 (1.5)	2.1 (2.6)	
Moderate (5–6 NRS) (25/15)	4.8 (3.2)	7.2 (4.9)		3.4 (3.0)	3.2 (3.9)	
Severe (7–10 NRS) (10/7)	7.4 (5.7)	7.8 (7.4)		7.9 (6.0)	7.9 (6.1)	
**Chronic/recur. musculoskeletal pain**	*F* = 8.1; *p* = 0.006	*F* = 8.4; *p* = 0.005	*p* ≥ 0.050	*p* ≥ 0.050	*p* ≥ 0.050	*p* ≥ 0.050
No (15/14)	1.2 (1.8)	1.6 (1.4)				
Yes (66/49)	4.3 (4.1)	5.5 (5.2)				
**Sleep problems** (ASSQ)	*F* = 8.2; *p* = 0.005	*p* ≥ 0.050	*F* = 5.6; *p* = 0.020	**F = 22.0; p < 0.001**	**F = 15.4; p < 0.001**	*p* ≥ 0.050
No/mild (67/51)	3.2 (3.6)		5.4 (6.6)	1.8 (1.8)	2.2 (2.7)	
Moderate/severe (14/12)	6.4 (4.4)		10.1 (7.4)	6.2 (5.6)	6.5 (5.7)	
**Symptoms of depression**	n/a	**F = 76.4; p < 0.001**	*p* ≥ 0.050	n/a	**F = 54.9; p < 0.001**	*p* ≥ 0.050
No or mild (PHQ-9 < 10) (72/59)		3.6 (3.5)			2.3 (2.7)	
Moderate or severe (PHQ-9 > = 10) (9/4)		14.6 (3.6)			13.0 (4.5)	
**Symptoms of generalised anxiety disorder**	**F = 78.8; p < 0.001**	n/a	*F* = 4.7; *p* = 0.033	**F = 57.3; p < 0.001**	n/a	*p* ≥ 0.050
No or mild (GAD-7 < 10) (68/59)	2.5 (2.4)		5.5 (6.6)	2.1 (2.2)		
Moderate or severe (GAD-7 > = 10) (13/4)	10.1 (4.4)		9.9 (7.5)	11.5 (5.0)		
**Symptoms of eating disorders**	*F* = 4.2; *p* = 0.044	*p* ≥ 0.050	n/a	*p* ≥ 0.050	*p* ≥ 0.050	n/a
No (EDE-QS < 15) (69/62)	3.3 (3.9)					
Yes (EDE-QS > = 15) (12/4)	5.8 (3.8)					
**Chronic/recurrent low mood**	**F = 19.5; p < 0.001**	**F = 11.0; p < 0.001**	*p* ≥ 0.050	**F = 34.6; p < 0.001**	**F = 17.1; p < 0.001**	*p* ≥ 0.050
No (73/56)	3.1 (3.3)	4.2 (4.3)		1.9 (1.9)	2.4 (2.7)	
Yes (8/7)	9.0 (5.8)	10.0 (7.3)		8.3 (6.2)	8.0 (7.1)	
**Chronic/recurrent generalised anxiety**	**F = 40.1; p < 0.001**	**F = 75.6; p < 0.001**	*F* = 5.1; *p* = 0.026	**F = 23.6; p < 0.001**	**F = 24.0; p < 0.001**	*p* ≥ 0.050
No (53/52)	2.1 (2.0)	2.3 (2.0)	5.0 (5.4)	1.8 (1.9)	2.1 (2.6)	
Yes (28/11)	6.9 (4.8)	9.5 (5.4)	8.5 (8.8)	6.5 (5.6)	7.4 (5.5)	
**Diagnosed with depression, anxiety, burnout or similar**	F = 6.0; *p* = 0.004	**F = 11.4; p < 0.001**	F = 3.5; *p* = 0.034	**F = 11.4; p < 0.001**	**F = 12.3; p < 0.001**	*p* ≥ 0.050
No (56/44)	2.8 (3.3)	3.4 (4.2)	5.0 (5.8)	2.0 (2.5)	2.2 (2.9)	
Previously (15/8)	6.1 (4.4)	7.9 (5.4)	7.0 (7.0)	2.6 (1.8)	3.4 (2.7)	
Currently (7/4)	6.0 (5.7)	10.1 (4.3)	11.4 (7.8)	9.3 (7.1)	10.5 (6.8)	
**Feel you should lose weight**	*p* ≥ 0.050	*p* ≥ 0.050	**F = 48.0; p < 0.001**	*p* ≥ 0.050	*p* ≥ 0.050	**F = 49.0; p < 0.001**
No (42/53)			2.2 (2.9)			2.4 (2.9)
Yes (37/13)			10.8 (7.5)			9.5 (4.6)
**Currently trying to lose weight**	*p* ≥ 0.050	*p* ≥ 0.050	**F = 53.5; p < 0.001**	*p* ≥ 0.050	*p* ≥ 0.050	**F = 64.5; p < 0.001**
No (52/53)			3.0 (3.5)			2.1 (2.4)
Yes (28/ 13)			12.3 (7.8)			10.2 (4.8)
**Diagnosed with eating disorder**	*F* = 3.9; *p* = **0**.026	*F* = 3.7; *p* = 0.030	**F = 14.8; p < 0.001**	*F* = 8.4; *p* = 0.005	n *p* ≥ 0.050	*p* ≥ 0.050
No (59/52)	3.1 (3.6)	3.9 (4.3)	4.7 (4.9)	2.3 (3.0)		
Previously (15/1)	5.1 (4.6)	7.0 (6.3)	7.9 (8.5)	11.0 (–)		
Currently (4/0)	7.5 (3.1)	8.3 (4.3)	21.3 (10.1)			
**Need for psychotherapeutic support**	**F = 6.6; p = 0.002**	**F = 7.4; p = 0.002**	**F = 8.8; p < 0.001**	F = 4.3; *p* = 0.017	*p* ≥ 0.050	*p* ≥ 0.050
No (27/26)	2.0 (2.0)	2.5 (3.3)	4.4 (0.8)	1.4 (1.7)		
Previously (30/21)	3.8 (4.2)	4.9 (5.2)	6.1 (1.1)	2.9 (2.7)		
Currently (23/16)	5.8 (4.5)	7.5 (5.1)	8.3 (1.7)	4.4 (5.1)		

*PHQ-9* depression module of the Patient Health Questionnaire, *GAD-7* Generalised Anxiety Disorder-7, *EDE-QS* Eating-Disorder-Examination-Questionnaire, *NRS* numeric rating scale, *ASSQ* Athlete Sleep Screening Questionnaire [37], *n/a* comparison not possible, *#* in the last 7 days

Results significant at *p* < 0.003 (Bonferroni corrected) are highlighted in bold

Multivariate regression analysis (Table 4) confirmed that neither the size or type of the companies, nor the age or rank of the dancers were related to the PHQ-9, GAD-7 and EDE-QS sum scores. The models for *PHQ-9* (*F* = 21.3; *p* < 0.001) and *GAD-7* (*F* = 19.3; *p* < 0.001) were statistically significant and explained 73.2% resp. 71.1% (corrected R^2^) of the variance. Musculoskeletal pain in the past seven days, sleep problems, the GAD-7 sum score and chronic or recurrent symptoms of low mood contributed significantly to the variability of the PHQ-9 sum score. Chronic or recurrent symptoms of generalised anxiety and the PHQ-9 sum score contributed significantly to the variability of the GAD-7 sum score (Table 4). The model for *EDE-QS* (*F* = 2.5; *p* < 0.003) was statistically significant but explained just 16.8% of the variance (corrected R^2^). The gender and body mass index contributed significantly to the variability of the EDE-QS sum score (Table 4).

**Table 4:** Results of the multivariate regression analysis on the depression (PHQ-9) [28], generalised anxiety disorder (GAD-7) [30] and the eating disorder (EDE-QS) [32] scores of female and male dancers

Variable (answer choices)	PHQ-9					GAD-7					EDE-QS				
	Beta	SE	Lower CI	Upper CI	*p*	Beta	SE	Lower CI	Upper CI	*p*	Beta	SE	Lower CI	Upper CI	*p*
**Gender** Female/male	− 0.122	0.667	− 1.444	1.201	0.856	− 0.252	0.849	− 1.937	1.432	0.767	− 5.186	1.826	− 8.808	− 1.563	**0.005**
**Size of the company** < 20/20–50/> 50 dancers	− 0.419	0.241	− 0.897	0.059	0.085	0.144	0.311	− 0.474	0.761	0.645	1.212	0.685	− 0.147	2.571	0.080
**Type of dance** Ballet/Contemporary/Revue	0.056	0.255	− 0.448	0.561	0.825	− 0.129	0.324	− 0.772	0.514	0.692	− 1.603	0.706	− 3.004	− 0.202	0.025
**Age of dancer** Years	0.044	0.039	− 0.033	0.121	0.257	− 0.098	0.049	− 0.195	− 0.002	0.046	− 0.002	0.111	− 0.222	0.218	0.988
**Rank of dancer** Principal dancer, soloist/Others	0.875	0.440	0.003	1.748	0.049	− 0.023	0.571	− 1.156	1.109	0.967	− 0.616	1.273	− 3.141	1.909	0.630
**Body mass index** Kg/m^2^	− 0.001	0.155	− 0.307	0.306	0.997	0.021	0.197	− 0.369	0.412	0.915	1.106	0.426	0.262	1.951	**0.011**
**Severity of musculoskeletal pain**^#^ Numerical rating scale 0–10	0.218	0.087	0.044	0.391	**0.014**	0.125	0.114	− 0.102	0.351	0.277	− 0.021	0.256	− 0.528	0.487	0.936
**Chronic/recur. musculoskeletal pain** No/Yes	− 0.158	0.486	− 1.123	0.807	0.746	0.950	0.613	− 0.266	2.165	0.124	− 0.201	1.384	− 2.945	2.543	0.885
**Sleep problems** (ASSQ) Sum score	0.201	0.070	0.061	0.341	**0.005**	− 0.106	0.093	− 0.290	0.078	0.256	0.064	0.208	− 0.348	0.477	0.758
**Symptoms of depression **(PHQ-9) Sum score					n/a	0.747	0.102	0.546	0.949	**0.000**	0.143	0.280	− 0.412	0.698	0.610
**Symptoms of generalised anxiety disorder** (GAD-7) Sum score	0.460	0.063	0.336	0.585	**0.000**					n/a	0.266	0.218	− 0.167	0.699	0.226
**Symptoms of eating disorders** (EDE-QS) Sum score	0.018	0.035	− 0.051	0.086	0.610	0.053	0.044	− 0.034	0.140	0.226					n/a
**Chronic/recurrent low mood** No/Yes	2.218	0.667	0.895	3.541	**0.001**	− 0.081	0.894	− 1.855	1.693	0.928	− 1.364	1.992	− 5.314	2.585	0.495
**Chronic/recurrent generalised anxiety** No/Yes	0.273	0.552	− 0.821	1.367	0.622	2.675	0.653	1.381	3.969	**0.000**	− 0.708	1.569	− 3.820	2.404	0.653
**Previously diagnosed with depression, anxiety, burnout or similar** No/Yes	0.109	0.516	− 0.914	1.132	0.833	0.962	0.650	− 0.328	2.252	0.142	− 0.956	1.464	− 3.859	1.947	0.515
**Previously diagnosed with eating disorder** No/Yes	0.527	0.618	− 0.699	1.754	0.396	− 0.263	0.790	− 1.831	1.304	0.740	2.855	1.742	− 0.600	6.311	0.104
**Need for psychotherapeutic support** No/Previously/Currently	0.361	0.253	− 0.140	0.863	0.156	− 0.231	0.325	− 0.874	0.413	0.479	1.330	0.714	− 0.087	2.746	0.065

Results significant at *p* < 0.017 (Bonferroni corrected) are highlighted in bold

*PHQ-9* depression module of the Patient Health Questionnaire, *GAD-7* Generalised Anxiety Disorder-7, *EDE-QS*: Eating-Disorder-Examination-Questionnaire, *NRS* numeric rating scale, *ASSQ* Athlete Sleep Screening Questionnaire [37], # in the last 7 days, *n/a* not included

## Discussion

To the best of our knowledge, this is the first study that assessed the prevalence of symptoms of depression, generalised anxiety disorders and eating disorders of professional dancers and compared the results to the general population of same gender and similar age. The study included 147 professional dancers from six opera houses or state theatres using well-established, validated questionnaires on symptoms of depression, generalised anxiety disorder and eating disorders (response rate 98%). The prevalence of at least moderate symptoms of depression was 11.1% in female and 6.4% in male dancers, of generalised anxiety disorder 16.0% in female and 6.4% in male dancers and of eating disorders 14.8% in female and 6.1% in male dancers. One in five (20.8%) dancers had at least moderate symptoms of either depression, generalised anxiety disorder or eating disorders. Uni- and multivariate analysis of potential determinants showed that the severity of symptoms of depression, generalised anxiety and eating disorders were independent of the size or type of the companies and of the age or rank of the dancers. The PHQ-9 and GAD-7 sum score were significantly related to severity of musculoskeletal pain in the past seven days and to chronic or recurrent symptoms of low mood and generalised anxiety. Female gender and BMI were the only significant variables to explain the variability of the EDE-QS sum score in the multivariate analyses.

### Prevalence of symptoms of depression

In the present study, the prevalence of moderate to severe depression symptoms was 11.1% in female and 6.4% in male dancers. A higher prevalence of depression in women than in men has previously been reported for various athlete populations [7, 10] and for the general population [29, 38]. Compared to a general population of the same gender and similar age [29], the prevalence was similar in male dancers, but about twice as high in female dancers. The results of the multivariate analysis of potential determinants showed that musculoskeletal pain in the past seven days, sleep problems, the GAD-7 sum score and chronic or recurrent symptoms of low mood contributed significantly to the variability of the PHQ-9 sum score. The association between acute pain and depression has been proven [39], and injury has been shown to be an important risk factor for mental health problems in athletes [40]. A significant association between symptoms of depression and with both a history of injury and active injuries has also been reported from collegiate dancers [23]. Therefore, especially dancers with acute or chronic injury/pain should be screened for mental health problems, as it has been recommended for elite athletes [4]. It is known that chronic or recurrent mental health problems or a previous diagnosis increases the risk of a new episode (e.g. [41]). Thus, it is important to diagnose and prevent mental health problems at an early stage. Attention should also be paid to sleep problems which could be symptom of mental health problems, but it can also lead to depression and anxiety [42]. A recent review concluded that improving sleep has beneficial effect on depression and anxiety [43].

### Prevalence of symptoms of generalised anxiety disorder

The prevalence of moderate to severe symptoms of generalised anxiety disorder was 16.0% in female and 6.4% in male dancers in the present study. A higher prevalence in women than in men is in accordance with the literature on elite athletes [5, 10] and the general population [31, 44]. In the present study, the prevalence in both female and male dancers was about twice as high as in a general population of the same gender and similar age. This seems to be in contrast to a review [8] that found no differences in the anxiety profiles of athletes and non-athletes. But the prevalence of generalised anxiety varies between sports and was highest in aesthetic sports [10]. Similar to aesthetic sports, dancers must fulfil a specific body image and are often judged not only by their performance but also by their physical appearance. A review on determinants of anxiety in elite athletes reported that pressure to perform, public scrutiny, career uncertainty or dissatisfaction and injury may precipitate or exacerbate anxiety disorders [8]. Thus, the specific employment situation in German companies (all contracts are limited to one year only) might have contributed to the high prevalence of anxiety symptoms of dancers in our study. Contracts renewals and selection for roles in performances are rarely built on objective criteria and are, therefore, not (or very little) within the dancer´s control. Further, most of the dancers in German companies are foreigners, i.e. they come from a different cultural background, do not speak German as native language or well enough to communicate with people outside the theatre and do not have families in the country or even on the same continent. Due to their daily schedule, it is difficult to find friends outside the working environments and the other dancers in the companies are also their competitor for role in the performances. Thus, the dancers lack a secure social environment which might (in addition to general dance-specific stressors [15, 16]) increase their risk of anxiety disorders and other mental health problems.

### Prevalence of symptoms of eating disorders

In the present study, the prevalence of eating disorders was 14.8% in female and 6.1% in male dancers. Thus, it was substantially higher than the 12-month prevalence of eating disorders in the general population (0.43%) [45] but similar to the prevalence of eating disorders in dancers reported previously (12.0%; 16.4% for ballet dancers [26]). The higher prevalence in women than in men is in accordance with findings in the general [45] and athlete populations [10, 12]. Dancers must fulfil a specific body image and are often judged not only by their performance but also by their physical appearance. In the present study, female gender and BMI were the only significant variables to explain the variability of the EDE-QS sum score. The average BMI of female dancers was 18.9 kg/m^2^, and in 42.9%, the BMI was below 18.5 kg/m^2^ (limit of for underweight), but still 47% of the female dancers thought that they should lose weight. Several intrapersonal (e.g. high perfectionism, low self-esteem, musculoskeletal pain), interpersonal (e.g. body image pressure) and dance cultural factors (e.g. physical ideals) have been shown to increase the risk of eating disorders in female dancers [16, 25]. Few studies analysed eating disorders in male (ballet) dancers [25]. In the present study, just one male dancer (1.8%) was diagnosed with an eating disorder previously or currently compared to almost a quarter of female dancers (24.3%) and no male dancer had an BMI below 18.5 kg/m^2^. However, the percentage of dancers who felt that they should change their weight and of those who tried to change their weight at the time of the screening was similar in both genders. The difference was that more male than female dancers thought they should gain weight (24.2% vs 2.5%) and fewer thought they should lose weight (19.7%vs 46.8%) resp. were trying to do so. Thus, male dancers seem as likely as female dancers to be dissatisfied with their body image and physical appearance but in a different way. While female dancers were aiming for a low body weight and a lean figure, male dancers wanted to shape their bodies to appear masculine, muscular and athletic. It is possible that male dancers have eating disorders as well, but these were not detected by the EDE-QS, since the questionnaire was developed and validated in mainly female populations [32, 33].

### Prevalence of at least one mental health problem

In accordance with previous findings in adolescent dance students [24], about 31.3% of dancers with eating disorders had co-occurring moderate or severe symptoms of depression and/or anxiety in the present study. More than a quarter (27.2%) of the female and 12.7% of the male dancers had moderate or severe symptoms of either depression, generalised anxiety or eating disorders at the time of the screening. In a representative sample of French athletes the prevalence of at least one psychopathology was lower (20.2%) in females and higher (15.1%) in males [10]. The higher percentage of mental health problems in female dancers might be caused by the higher prevalence of eating disorders in this group compared to female athletes in general.

### Need for psychotherapeutic support

In the present study, about a quarter of the dancers stated that they “wanted or needed support from a psychotherapist/psychologist for personal or mental health problems” at the time of the screening without difference between genders. This is substantially lower than reported from collegiate dancers (37.4%) [23] but higher than in elite Aquatic athletes (15.9%) [46], elite amateur golfers (17.9%) [47] and in elite female football players (15.7%, 16.0%) [48, 49]. The high prevalence in collegiate dancers might be due to their age, stress from academic and dance examinations, and the data collection during COVID-19 pandemic (summer 2021) [23]. A likely explanation for the higher percentage in our population compared to athletes is the higher prevalence of mental health problems as outlined above.

Almost 70% of the dancers with at least moderate symptoms of depression, but less than half (43.8%) of the dancers with an eating disorder and a third (3 of 9) of the dancers with a severe underweight (BMI < 17.5) stated that they wanted or needed psychotherapeutic support at the time of the screening. It is likely that stigma, denial and the demand for leanness in the ballet environment contributed to this result.

### Strength and limitations

Since participation in the health screening was voluntary not all dancers employed at the six companies could be included in the study, however of those participating 98% filled in the PHQ-9 and GAD-7, all the EDE-QS. The participation rate varied substantially between companies (42.6% in large, 76.9% in medium and 91.3% in small companies), and the mean PHQ-9, GAD-7 and EDE-QS scores were similar in companies of different size. Thus, it seems very likely that the dancers in the present study are similar to all dancers of the included companies.

The PHQ-9 and GAD-7 have been recommended as disorder-specific screening questionnaires by the IOC [3]. The EDE-QS has recently been highly recommended over the BEDA-Q for assessing eating disorders in athlete populations [50]. However, questionnaires should only serve as screening tools, and a diagnosis should be based on diagnostic interviews by qualified healthcare professionals. It cannot be ruled out that some answers to the questionnaires were biased due to stigma, denial, low mental health literacy or fear of potential consequences [51], although confidentiality was assured, but this limitation also applies to almost all studies on mental health in elite sports [4, 52].

Age- and gender-specific references values from representative surveys were used for comparison of the PHQ- and GAD-7 data with the general population. These surveys were more than 10 years old; however, it has been demonstrated that the age-standardised prevalence of depression and anxiety disorders has not changed from 1990 to 2019 [19, 53], and that mental health symptoms were comparable to pre-pandemic levels by mid-2020 among most population sub-groups and symptom types [54]. Our study was conducted in August 2022. No newer representative survey using these questionnaires in a similar age group and no representative survey using the EDE-QS in the general population were found in the literature for comparison.

Only companies of German state theatres or opera houses were included but of different size and dance style. It was shown that these variables had no significant effect on the mean depression, generalised anxiety disorder or eating disorders scores. Some subgroups in Table 3 are very small. Small sample sizes reduce the statistical power and increase the risk of a type-2 error, i.e. true differences might not be detected. However, separate analysis of female and male dancers seemed appropriate given the gender differences in PHQ-9, GAD-7 and EDE-QS. The analysis of potential determinants used multiple testing, but this was controlled by Bonferroni correction and in addition a multivariate regression analysis was performed. Due to the cross-sectional design of the present study, no cause-effect relations could be assessed.

## Conclusion

The present study showed that the prevalence of symptoms of generalised anxiety and eating disorders was higher in professional dancers than in the general population of similar age and same gender. One in five dancers (20.8%) had moderate or severe symptoms of either depression, generalised anxiety or eating disorders. Therefore, mental health should be part of the routine (pre-season) health screening [4]. Further, mental health awareness and literacy [55] should be raised, stigma reduced and low-threshold access to adequate treatment [51] provided. Prospective studies are required to analyse risk factors and to develop and evaluate prevention strategies for ill mental health of professional dancers.

### Supplementary Information

**Additional file 1.** Dancer´s Health Questionnaire (short version).

## Data Availability

Due to confidentiality reasons, no data can be shared.

## Abbreviations

ANOVA: Analysis of variance
ASSQ: Athlete Sleep Screening Questionnaire
BEDA-Q: Brief Eating Disorder in Athletes Questionnaire
BMI: Body mass index
EDE-QS: Eating-Disorder-Examination-Questionnaire
GAD-7: Generalised Anxiety Disorder-7
IOC: International Olympic Committee
LEA: Low energy availability
NRS: Numeric rating scale
PHQ-9: Depression module of the Patient Health Questionnaire
RED-S: Relative energy deficiency in sport
SD: Standard deviation
SPSS: Statistical Package for Social Sciences
