# Supplementary material for: Behind the Curtain: Prevalence of Symptoms of Depression, Generalised Anxiety and Eating Disorders in 147 Professional Dancers from Six Opera Houses or State Theatres
Source: Sports Med Open. 2023 Sep 30;9:92. doi: 10.1186/s40798-023-00638-9 (PMC10542047; doi:10.1186/s40798-023-00638-9)
Supplement: Supplementary file 1 — Additional file 1. Dancer´s Health Questionnaire (short version). [file 40798_2023_638_MOESM1_ESM.docx]

**Journal of Sports Medicine Open**

**Behind the curtain –
Prevalence of symptoms of depression, generalised anxiety and eating disorders in 147 professional dancers
from six opera houses or state theatres**

Astrid Junge^1^, Anja Hauschild^1,2^

^1^ Center for Health in Performing Arts, Medical School Hamburg (MSH), Hamburg, Germany

^2^ Center for Rehabilitation and Sports Medicine, BG Klinikum Hamburg, Hamburg, Germany

shortened questionnaire

Only questions analysed in the publication “Behind the curtain – Prevalence of symptoms of depression, generalised anxiety and eating disorders in 147 professional dancers from six opera houses or state theatres” by Junge and Hauschild are presented.

For the full version of the questionnaire used in the Dancers Health Project, please contact the authors via Astrid.Junge@medicalschool-hamburg.de


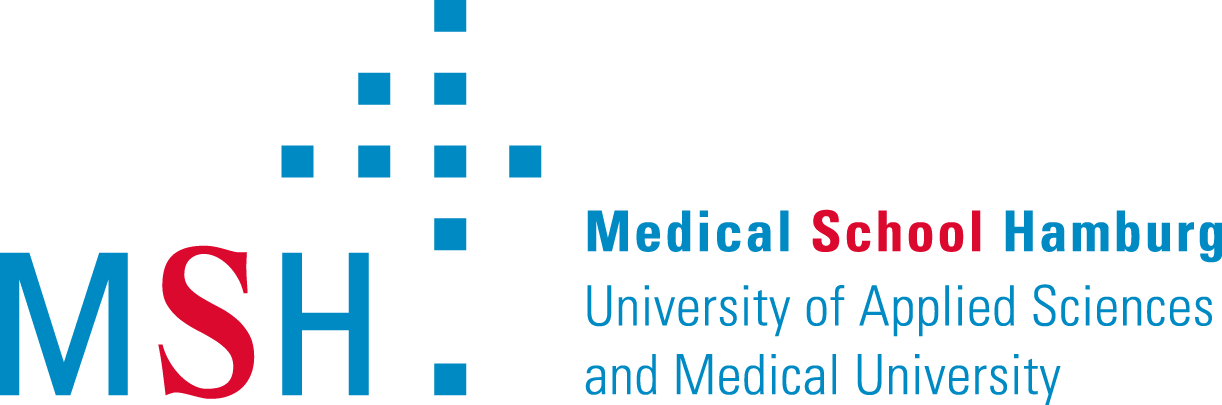


**Dancer´s Health –**

**Epidemiology, risk factors and prevention of injuries and complaints
of professional dancers**

The first questions relate to **you, your dancing experiences and practice**

Which **gender** are you?

◻ female ◻ male ◻ others

How **old** are you? _________years

For how many **years** did you participate in **a professional dance education programme**? _____

At what **age** did you start to **dance in a professional company**? _________ years

When did you **join** your **current dance company**? ________ (please state year)

Which **rank** do you currently hold?

◻ Principal dancer

◻ Soloist

◻ Semi-Soloist / Coryphées / Corps de Ballet

◻ Eleve

The next questions refer to your general **medical history**

Please state your current **body height** and **weight**: _____cm ______kg

Do you **feel you should** lose or gain weight?

◻ no ◻ yes, lose weight ◻ yes, gain weight

Are you currently **trying to** **lose or gain weight**?

◻ no ◻ yes, trying to lose weight ◻ yes, trying to gain weight

Do you eat a **special diet** or exclude any foods?
◻ no

◻ vegetarian ◻ vegan ◻ gluten-free ◻ low carbohydrate ◻ low fat

◻ other, please specify

Do you take **food supplements** (i.e. vitamins, minerals, or protein as pills or drinks)?

◻ no

◻ vitamins ◻ minerals ◻ proteins ◻ others, please specify ____________

Have you ever been **diagnosed** with and/or treated for the following **illnesses** or complaints?

|  | no | yes, previously | yes, currently |
| --- | --- | --- | --- |
| allergy (food, animals, medication, etc.) | ◻ | ◻ | ◻ |
| asthma | ◻ | ◻ | ◻ |
| migraine | ◻ | ◻ | ◻ |
| iron deficiency / anaemia | ◻ | ◻ | ◻ |
| depression, anxiety, burnout or similar | ◻ | ◻ | ◻ |
| eating disorder | ◻ | ◻ | ◻ |
| others, please specify | ◻ | ◻ | ◻ |
|  | | | |

Do you have any **other chronic** or **recurring complaints?**

◻ no

◻ headache ◻ low mood

◻ abdomen pain ◻ general anxiety

◻ fatigue, lack of energy ◻ performance anxiety / stage fright

◻ concentration problems

◻ dizziness

◻ others, specify_________________________________________________________________

Have you **ever wanted** or **needed** support from a psychotherapist / psychologist for personal or mental health problems?

◻ no ◻ yes, **previously**  ◻ yes, **currently**

The following questions refer to your **current complaints**

**How severe** have the following complaints / symptoms been **in the last 7 days?**

*Please rate each symptom on the scale from “not at all” (0) to “worst imaginable” (10).*

|  | not worst  at all imaginable |
| --- | --- |
| musculo-skeletal pain | 0        1        2        3        4        5        6        7        8        9        10 |

The dancers also answered the following questionnaires:

PHQ-9: **depression module of the Patient Health Questionnaire**

Kroenke K, Spitzer RL, Williams JB. The PHQ-9: validity of a brief depression severity measure. J Gen Intern Med 2001;16:606–13

GAD-7: **Generalized Anxiety Disorder-7**

Spitzer RL, Kroenke K, Williams JBW, Löwe B. A brief measure for assessing generalized anxiety disorder: the GAD-7. Arch Intern Med 2006;166:1092–7.

EDE-QS: **Eating-Disorder-Examination-Questionnaire**

Gideon N, Hawkes N, Mond J, Saunders R, Tchanturia K, Serpell L. Development and psychometric validation of the EDE-QS, a 12 item short form of the eating disorder examination questionnaire (EDE-Q). PLoS One 2016;11(5):e0152744.

ASSQ: **Athlete Sleep Screening Questionnaire**

Bender AM, Lawson D, Werthner P, Samuels CH. The clinical validation of the athlete sleep screening questionnaire: an instrument to identify athletes that need further sleep assessment. Sports Med Open 2018;4(1):23
